# Estratificação de Risco e Avaliação da Atividade Simpática Cardíaca Utilizando Imagem Miocárdica com [^123^I] MIBG na Denervação Renal

**DOI:** 10.36660/abc.20201253

**Published:** 2022-02-14

**Authors:** Joana Delgado-Silva, Ana Paula Moreira, Gracinda Costa, Lino Gonçalves

**Affiliations:** 1 Faculdade de Medicina Universidade de Coimbra Coimbra Portugal Faculdade de Medicina (FMUC), Universidade de Coimbra, Coimbra – Portugal; 2 Departamento de Cardiologia Centro Hospitalar e Universitário de Coimbra Coimbra Portugal Departamento de Cardiologia, Centro Hospitalar e Universitário de Coimbra (CHUC), Coimbra – Portugal; 3 Departamento de Medicina Nuclear Centro Hospitalar e Universitário de Coimbra Coimbra Portugal Departamento de Medicina Nuclear, Centro Hospitalar e Universitário de Coimbra (CHUC), Coimbra – Portugal; 4 Instituto de Ciências Nucleares Aplicadas à Saúde Universidade de Coimbra Coimbra Portugal Instituto de Ciências Nucleares Aplicadas à Saúde (ICNAS), Universidade de Coimbra, Coimbra – Portugal; 5 Instituto de Investigação Clínica e Biomédica de Coimbra Faculdade de Medicina Universidade de Coimbra Coimbra Portugal Instituto de Investigação Clínica e Biomédica de Coimbra (iCBR), Faculdade de Medicina, Universidade de Coimbra, Coimbra – Portugal

**Keywords:** Hipertensão Resistente, Rim/denervação, Sistema Nervoso Simpático, Miocárdio/diagnóstico por Imagem, Cintilografia, 3-Iodobenzilguanidina

## Abstract

A hiperativação do sistema nervoso simpático desempenha um papel central na fisiopatologia da hipertensão. O objetivo deste estudo foi avaliar a atividade simpática cardíaca e investigar o papel da cintigrafia miocárdica com metaiodobenzilguanidina com ^123^I ([^123^I] MIBG) na estratificação de risco cardiovascular de pacientes com hipertensão resistente tratados com denervação renal (DR). Dezoito pacientes foram incluídos neste estudo prospectivo (média de idade de 56 ± 10 anos, 27,8% mulheres). Ecocardiograma transtorácico, análise geral do sangue e cintilografia miocárdica com [(^123^I) MIBG] foram realizados antes e seis meses após a DR. Um paciente era considerado respondedor (R) se uma diminuição ≥ 5 mmHg na pressão arterial sistólica (PAS) média ambulatorial fosse observada no seguimento de seis meses. 66,7% dos pacientes foram R (diminuição na PAS de 20,6 ± 14,5 mmHg, vs. menos 8 ± 11,6 mmHg em não-respondedores (NR), p = 0,001). A relação coração-mediastino (RCM) inicial foi significativamente menor na linha basal no grupo R (1,6 ± 0,1 vs. 1,72 ± 0,1, p <0,02), mas semelhante em seis meses. Considerando os dois momentos no tempo, o grupo R teve valores iniciais de RCM mais baixos do que o grupo NR (p <0,05). Tanto o RCM tardio quanto a taxa de *washout* foram idênticos e nenhuma correlação significativa entre a resposta à DR ou qualquer índice de imagem com MIBG foi encontrada. A denervação renal efetivamente reduziu a pressão arterial na maioria dos pacientes, mas a imagem com [^123^I] MIBG não foi útil na previsão da resposta. Entretanto, houve evidência de *overdrive* do sistema nervoso simpático e, tanto a RCM inicial quanto tardia estavam reduzidas em geral, provavelmente colocando essa população em um risco maior de eventos adversos.

## Introdução

A hipertensão (HT) há muito é reconhecida como uma das principais causas de morte cardiovascular e hospitalizações.^1^ De acordo com as diretrizes atuais, a HT é definida como resistente quando a terapia farmacológica otimizada com três medicamentos anti-hipertensivos, incluindo um diurético, é incapaz de efetivamente reduzir a pressão arterial (PA) sistólica e diastólica para <140mmHg e <90mmHg, respectivamente. Acredita-se que sua prevalência seja em torno de 5 a 15%.^2^ O sistema nervoso simpático (SNS) e seu envolvimento na regulação circulatória foram demonstrados pela primeira vez no século 19, mostrando que a estimulação dos nervos renais elevava a PA.^3^ De acordo com esse conhecimento, procedimentos invasivos direcionados ao SNS foram desenvolvidos no início / meados do século 20, mas foram descontinuados devido ao aumento dos efeitos colaterais e mortalidade.^4^ Desde então, o esclarecimento dos mecanismos pelos quais o SNS leva ao descontrole da PA conduziu ao desenvolvimento de um procedimento percutâneo minimamente invasivo que demonstrou reduzir a atividade simpática renal e central.^5^ A denervação renal (DR) tem sido objeto de extensa investigação nos últimos anos e os últimos estudos randomizados de segunda geração demonstraram eficácia na redução da PA, mas também segurança, em coortes de pacientes com diferentes níveis de risco cardiovascular.^6^ A metaiodobenzilguanidina marcada com ^123^I ([^123^I] MIBG) é um análogo da norepinefrina (NE), marcada com iodo-123, que compartilha o mesmo mecanismo de captação nos nervos pré-sinápticos. Após a captação, é transportada para as vesículas de armazenamento de catecolaminas e, por não ser metabolizada, permite a caracterização da atividade simpática cardíaca e integridade neuronal através da aquisição de imagens planas por câmaras gama. Ao analisar as imagens, dois parâmetros semiquantitativos são calculados: a relação coração-mediastino (RCM) inicial e tardia e taxa de *washout* (WR, do inglês *washout rate* ). Aumentos na concentração de [^123^I] MIBG na fenda sináptica se traduziram em WR aumentada e RCM diminuída.

A longo prazo, um SNS em hiperativação crônica leva a uma falta significativa de função / redução dos transportadores de NE (aumentando a concentração sináptica de NE) e à exaustão das vesículas de armazenamento de NE. A quantidade excessiva de catecolaminas cardíacas promove fibrose, necrose de cardiomiócitos e predispõe a eventos arrítmicos graves. As imagens iniciais obtidas com a cintigrafia miocárdica com [^123^I] MIBG (MIBG-C) caracterizam a captação intersticial, refletindo a integridade dos neurônios pré-sinápticos, enquanto as imagens tardias representam a distribuição dos terminais nervosos simpáticos, refletindo a função neuronal. A WR representa a capacidade do miocárdio de reter MIBG e depende da integridade neuronal e do grau de atividade simpática.^7^

O objetivo deste estudo foi avaliar a atividade simpática cardíaca e investigar o papel da [^123^I] MIBG-C miocárdica na estratificação de risco cardiovascular de pacientes com HT resistente tratados com DR.

## Métodos

Incluímos, neste estudo prospectivo de centro único, 18 pacientes consecutivos com HT resistente tratados com DR, de maio de 2014 a outubro de 2017. Um histórico médico abrangente foi registrado em todos os pacientes e HT secundária não tratada foi excluída. A adesão à terapia medicamentosa foi confirmada por ingestão testemunhada (os pacientes foram internados na enfermaria de cardiologia por um período de 24 horas). Os critérios de exclusão incluíram eventos cardiovasculares adversos importantes recentes, displasia fibromuscular, angioplastia renal anterior, taxa de filtração glomerular <45mL / min / 1,73m^2^, HT secundária não tratada e pseudo-HT (90 pacientes excluídos). Foram incluídos pacientes com PA sistólica média >135 mmHg (monitoração ambulatorial da pressão arterial - MAPA). Um total de 108 pacientes com suspeita de hipertensão resistente verdadeira foram avaliados no ambulatório e 90 pacientes foram excluídos, de acordo com os critérios descritos. Todos os pacientes foram submetidos a uma avaliação clínica completa, eletrocardiograma, ecocardiograma transtorácico, perfil hematológico e bioquímico padrão e MIBG-C, tanto no início do estudo quanto no seguimento de seis meses. Para o procedimento de DR, o sistema multieletrodo EnligHTN (St. Jude Medical, MN, EUA) foi utilizado em 33,3% dos casos e o cateter multieletrodo Symplicity Spyral (Medtronic Inc., Santa Rosa, CA, EUA) em 66,7%. Todos os pacientes receberam sedação e analgesia consciente, e a hemostasia da artéria femoral foi realizada com um dispositivo de fechamento vascular. Antes da MIBG-C, os pacientes foram pré-tratados com solução de Lugol para bloqueio da tireoide (equivalente a 130 mg de iodo para adultos) ou 500 mg de perclorato de potássio se o paciente fosse alérgico a iodo. Em seguida, uma injeção intravenosa de 185 MBq de [^123^I] MIBG foi administrada e as imagens planares do tórax foram adquiridas com uma câmara gama de duas cabeças, quinze minutos (imagem inicial) e quatro horas (imagem tardia) após a administração do radiofármaco. A captação de MIBG foi semiquantificada pelo cálculo da RCM, após traçar as regiões de interesse (ROIs, do inglês *regions of interest* ) sobre o coração (incluindo a cavidade) e o mediastino superior (evitando a glândula tireoide) na projeção anterior plana. As contagens médias por pixel no miocárdio foram divididas pelas contagens médias por pixel no mediastino. A WR miocárdica das imagens iniciais para as tardias também foi calculada e expressa em porcentagem, sendo a taxa de redução da contagem miocárdica ao longo do tempo, entre as imagens iniciais e tardias (normalizadas para a atividade mediastinal). Nenhum dos medicamentos prescritos foi interrompido para a realização da MIBG-C, devido à alta probabilidade de eventos adversos e, consequentemente, por questões éticas. A resposta à DR foi definida se uma queda na PA sistólica média na MAPA ≥ 5 mmHg fosse observada em seis meses e os pacientes foram divididos em dois grupos adequadamente.

As variáveis categóricas foram caracterizadas pela determinação das frequências absolutas e relativas e as variáveis numéricas pelas médias e desvios-padrão. A normalidade da distribuição foi verificada e um valor de p <0,05 foi considerado significante. As comparações entre os grupos em relação às variáveis categóricas foram realizadas através do Teste de Qui-Quadrado. Em relação às variáveis contínuas, o teste U de Mann-Whitney foi utilizado para comparar dois grupos. Um modelo linear geral para medidas repetidas foi aplicado para analisar a variância de cada parâmetro, medido antes e depois da DR em cada indivíduo de dois grupos diferentes, ‘respondedor’ e ‘não respondedor’. As análises estatísticas foram realizadas utilizando o software SPSS 19.0®, com nível de significância de 5% para o teste de hipóteses. Este estudo foi aprovado pela Comitê de Ética da Faculdade de Medicina de Coimbra e todos os pacientes assinaram o termo de consentimento livre e esclarecido.

## Resultados

Dezoito pacientes (média de idade 56 ± 10 anos, 27,8% mulheres) foram incluídos neste estudo. Doze pacientes eram ‘respondedores’ (R, 66,7%) e seis ‘não respondedores’ (NR, 33,3%). Não foram observadas diferenças significativas entre os grupos em relação às características basais. A DR foi bem tolerada por todos os pacientes e nenhuma complicação periprocedimento foi detectada. O tempo de fluoroscopia foi significantemente maior no grupo NR (16,3 ± 5,5 vs. 26,5 ± 18,6 minutos, p <0,04). No seguimento de 6 meses, um paciente apresentou edema agudo de pulmão, sendo diagnosticado com estenose renal, tratado com sucesso com angioplastia. Este paciente era um ‘respondedor’ visto que uma queda ≥ 5 mmHg na PA sistólica média da MAPA foi observada 15 dias após a angioplastia. Uma queda de 20,6 ± 14,5 mmHg na PA sistólica média da MAPA foi observada no grupo R (vs. -8 ± 11,6 mmHg no grupo NR, p = 0,001). Embora a PA sistólica de consultório não tenha sido considerada para a resposta, devido ao possível ‘efeito do avental branco’, uma queda também foi observada no grupo R (29,2 ± 8,4 mmHg) vs. o grupo NR (13 ± 13,4 mmHg) (p = 0,09). Nenhum efeito colateral, como hipotensão ortostática, distúrbios eletrolíticos ou insuficiência renal, foi observado no seguimento de médio prazo. Os achados na ecocardiografia transtorácica (em relação à função diastólica, espessura da parede ou função sistólica biventricular) não diferiram significantemente entre os dois grupos, seja na linha basal ou na avaliação de 6 meses. As características da linha basal e relacionadas ao procedimento nos grupos de ‘respondedores’ e ‘não respondedores’ em geral, são mostradas na Tabela 1 .

**Tabela 1:** Características basais e relacionadas ao procedimento, MAPA basal e evolução de 6 meses e parâmetros da cintigrafia com MIBG basal e de seguimento de 6 meses, em geral, grupos ‘respondedores’ e ‘não respondedores’

	Geral	R (n=12)	NR (n=6)	p valor
**Características gerais basais**				
Média de idade (A)	56 ± 10	58,4 ± 9,8	51,3 ± 10,3	0,17 (ns)
Sexo feminino (%)	27,8	16,7	50	0,14 (ns)
Diagnóstico de HT (A)	19 ± 7,9	19,8 ± 8,7	17,5 ± 6,2	0,57 (ns)
Dislipidemia (%)	88,9	83,3	100	0,29 (ns)
Diabetes (%)	44,4	41,7	50	0,73 (ns)
Tabagismo ativo (%)	27,8	16,7	50	0,14 (ns)
IMC (Kg/m2)	29,7 ± 4,1	29,5 ± 4	30 ± 4,7	0,84 (ns)
Apneia do sono (%)	66,7	66,7	66,7	1 (ns)
Número de medicamentos para HT (n ± DP)	5,2 ± 1,2	5,2 ± 1,5	5,3 ± 0,5	0,79 (ns)
Espironolactona (%)	61,1	66,7	50	051 (ns)
Bloqueadores do canal de cálcio (%)	100	100	100	----
Betabloqueadores (%)	77,8	75	83,3	0,7 (ns)
Inibidores da ECA/BRAs (%)	94,4	91,7	100	0,48 (ns)
Diuréticos (%)	94,4	91,7	100	0,48 (ns)
Bloqueadores alfa-2 (%)	61,1	58,3	66,7	0,74 (ns)
Creatinina basal (mg/dl)	0,88 ± 0,2	0,9 ± 0,2	0,7 ± 0,2	0,56 (ns)
**Eco basal e após 6M**				
Fração de ejeção basal(%)	59 ± 9	59 ± 9	59 ± 8	0,94 (ns)
Fração de ejeção 6M (%)	58 ± 9	56 ± 9	62 ± 9	0,21 (ns)
Espessura do SIV basal (mm)	12,4 ± 3,7	13,4 ± 4,2	10,5 ± 1,4	0,12 (ns)
Espessura do SIV 6M (mm)	12,9 ± 2,6	13,4 ± 3	12 ± 1,3	0,29 (ns)
Espessura da PP basal (mm)	10,9 ± 1,9	11,5 ± 2	9,7 ± 1,2	0,06 (ns)
Espessura da PP 6M (mm)	10,2 ± 2,3	10,5 ± 2,5	9,7 ± 1,6	0,48 (ns)
Volume AE basal (mL/m^2^)	56,4 ± 19,5	53,8 ± 14,8	61,8 ± 11,3	0,43 (ns)
Volume AE 6M (mL/m^2^)	56 ± 22,3	59 ± 23	51 ± 22	0,51 (ns)
E / E ‘ basal	11,1 ± 3,5	9,9 ± 3	13,6 ± 3,2	0,02
E/E’ 6M	11 ± 3,5	10,9 ± 4	11 ± 2,4	0,95 (ns)
**MAPA basal e 6M**				
PAS média basal (mmHg)	154,6 ± 11,7	154,5 ± 11,4	154,8 ± 13,5	0,96 (ns)
Queda na PAS 6M (mmHg)	11 ± 19,2	20,6 ± 14,5	-8 ± 11,6	0,001
PAD média basal (mmHg)	90,7 ± 14	88,2 ± 13,5	95,7 ± 14,8	0,29 (ns)
Queda na PAD 6M (mmHg)	6,3 ± 9	10,4 ± 7,1	-1,8 ± 6,5	0,004
Frequência cardíaca basal (bpm)	71 ± 10	70 ± 9	73 ± 14	0,67 (ns)
Frequência cardíaca 6M	70 ± 10	68 ± 9	76 ± 11	0,44 (ns)
**Denervação renal**				
Número de ablações (n ± DP)	27,2 ± 7,7	28,7 ± 8,1	24,2 ± 6,3	0,25 (ns)
Tempo de fluoroscopia (min)	19,3 ± 11,4	16,3 ± 5,5	26,5 ± 18,6	<0,04
**Cintigrafia cardíaca com MIBG**				
RCM basal 15 min	1,63 ± 0,11	1,59 ± 0,10	1,72 ± 0,08	<0,02
RCM 6M 15 min	1,64 ± 0,12	1,61 ± 0,10	1,70 ± 0,14	0,14 (ns)
RCM basal 4 horas	1,60 ± 0,11	1,59 ± 0,10	1,64 ± 0,14	0,22 (ns)
RCM 6M 4 horas	1,60 ± 0,16	1,59 ± 0,12	1,63 ± 0,24	0,64 (ns)
WR basal	22,7 ± 18,6	17,9 ± 10	32,2 ± 28,2	0,13 (ns)
WR 6M	25,9 ± 16,4	25,4 ± 17,9	27 ± 14,2	0,86 (ns)

*A: anos; HT: hipertensão; IMC: índice de massa corporal; ECA: enzima de conversão da angiotensina; BRAs: bloqueadores do receptor da angiotensina; M: meses; SIV: septo intraventricular; PP: parede posterior; AE: átrio esquerdo; MAPA: monitoração ambulatorial da pressão arterial; PAS: pressão arterial sistólica; PAD: pressão arterial diastólica; bpm: batimentos por minuto; MIBG: metaiodobenzilguanidina marcada com ^123^I; RCM: relação coração-mediastino; WR: taxa de washout. Os resultados são exibidos como média ± desvio padrão (DP).*

**Figura 1:**
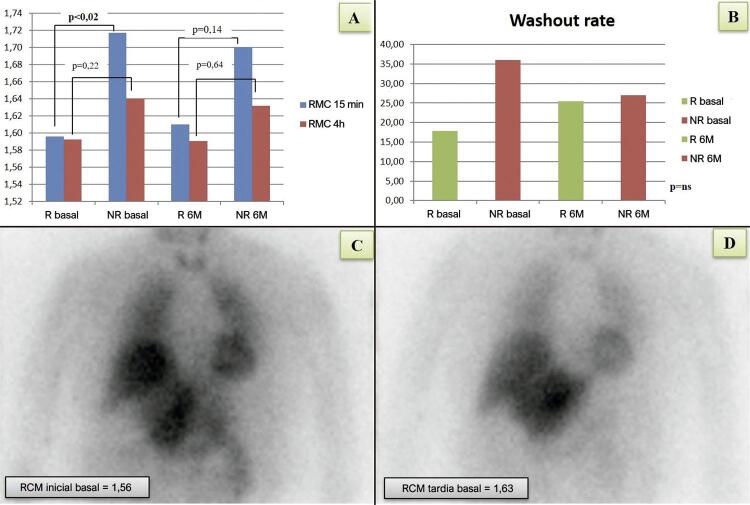
– Cintigrafia miocárdica com MIBG em pacientes submetidos à denervação renal (DR). (A) Relação coração-mediastino (RMC) inicial (15 minutos) e tardia (4 horas) na linha basal e seis meses (6M) após a DR, em ‘respondedores’ (R) vs. ‘não respondedores’ (NR) – a RMC inicial foi significantemente mais baixa em R, na linha basal; (B) Taxa de washout na linha basal e seis meses após a DR em R vs. NR; (C) e (D) cintilografia com MIBG, projeção anterior do tórax, na linha basal, em um respondedor, aos 15 minutos (C) e após quatro horas (D).

A RCM inicial foi significantemente menor na linha basal do grupo R (1,6 ± 0,1 vs. 1,72 ± 0,1, p <0,02, IC95% 1,6-1,71), mas não foi estatisticamente diferente do grupo NR em seis meses. Considerando os dois períodos de tempo, o grupo R teve valores iniciais de RCM mais baixos do que o grupo NR (p <0,05, IC 95% 1,58-1,69). Em relação à RCM e WR tardias, as diferenças antes e depois da DR não foram significantes entre os grupos. Nenhuma correlação significativa entre a resposta à DR ou qualquer índice de imagem com [^123^I] MIBG foi encontrada, seja na linha basal ou no seguimento ( Tabela 1 , Figura 1 e Tabela complementar 1 ).

## Discussão

O objetivo do nosso estudo foi determinar se a DR teve algum impacto na atividade simpática miocárdica, e também avaliar a segurança do procedimento, uma vez que uma diminuição significativa na RCM posteriormente, poderia significar interrupção da via simpática. Verificamos uma redução significativa da PA seis meses após a DR, em 66,7% dos pacientes, o que está de acordo com a eficácia relatada da técnica. Não foram relatados problemas de segurança, exceto em um paciente que foi diagnosticado com estenose da artéria renal seis meses após a DR, provavelmente devido à aplicação de radiofrequência próxima a uma placa aterosclerótica não significativa. Determinamos que os respondedores tinham uma RCM inicial basal significativamente mais baixa, o que poderia ser devido à integridade neuronal diminuída, mas nenhuma mudança significante foi observada após seis meses.

A CMR tardia foi semelhante em ambos os grupos, mas reduzida em comparação aos valores relatados em indivíduos normais (valores normais relatados: 2,2 ± 0,3,^5^ valores de referência locais 1,9-2,8), tanto na linha basal quanto no seguimento de seis meses, traduzindo-se em um comportamento de hiperatividade simpática mantida mesmo após a DR, e provavelmente associada a um maior risco de eventos. A WR também foi estatisticamente semelhante em ambos os grupos. Entretanto, a WR estava significantemente aumentada em geral, em comparação com indivíduos normais (valores médios normais relatados de 10 ± 9%,^5^ valores de referência locais 8,5-9,6%), sendo que essa discrepância foi mais evidente em não respondedores na linha basal, devido a um possível overdrive simpático.

O SNS é um sistema extremamente complexo, com implicações clínicas em estados fisiológicos e patológicos. É caracterizado por múltiplos níveis de ação que envolvem regulação central, transmissão ganglionar, liberação e recaptação de norepinefrina e resposta de receptores adrenérgicos.^8^ Dessa forma, não existe um método preciso para avaliar a atividade simpática global e regional, sendo que cada técnica tem seus pontos fortes e limitações.

O efeito da DR na atividade simpática foi descrito anteriormente. Krum et al.,^9^ relataram uma redução de 47% na liberação de noradrenalina dos nervos simpáticos renais bilateralmente, após a DR, utilizando o método de diluição de isótopos na noradrenalina renal ( *spillover* ). A MIBG-C foi realizada em pequenas coortes de pacientes com DR para avaliar a atividade simpática, mas os resultados foram bastante divergentes, relatando diminuições na WR,^5^ aumentos na RCM^10^ tardia ou nenhuma alteração.^11^ Esse método de imagem também tem sido considerado útil para avaliar a atividade simpática cardíaca no contexto de insuficiência cardíaca, podendo estimar o prognóstico e a resposta ao tratamento. De fato, no estudo ADMIRE-HF, uma taxa significantemente menor de eventos e morte cardíaca foi observada em pacientes com um RCM tardia ≥ 1,6.^12^

O que não está claro em nosso estudo é que os não respondedores apresentaram evidência de aumento da atividade do SNS, por que eles não responderam clinicamente à DR? Haveria outros fatores/sistemas suplantando a contribuição do SNS na fisiopatologia da HT? Além disso, nenhuma das taxas avaliadas mostrou alteração significante no seguimento, traduzindo uma ausência de distúrbios deletérios dos nervos simpáticos e, nenhum dos parâmetros da MIBG avaliados foram úteis para prever a resposta à DR.

### Limitações

Nosso estudo tem algumas limitações. Em primeiro lugar, este é um estudo de centro único e o número de pacientes inscritos é pequeno. Em segundo lugar, não havia grupo controle. Terceiro, dois sistemas de denervação diferentes foram utilizados, embora ambos fossem do tipo multieletrodo. Por fim, o estudo não foi randomizado e, embora o especialista em medicina nuclear fosse altamente experiente, não houve validação interna ou externa dos resultados.

## Conclusões

Neste estudo, demonstramos que a denervação renal reduziu significativamente a pressão arterial em uma porcentagem significante de pacientes, mas não houve evidência de redução da atividade simpática cardíaca observada pela cintigrafia miocárdica com [^123^I] metaiodobenzilguanidina. Nenhum dos parâmetros de imagem foi útil para prever a resposta à denervação renal. No entanto, tanto a relação coração-mediastino inicial quanto a tardia mostraram-se reduzidas, em comparação com a população em geral, provavelmente colocando essa população em maior risco de eventos. Estudos em larga escala são necessários para determinar a validade desse método na avaliação dos efeitos da denervação renal cardíaca.

## * Material suplementar

Para informação adicional, por favor, clique aqui .
