# Supplementary material for: Estratificação de Risco e Avaliação da Atividade Simpática Cardíaca Utilizando Imagem Miocárdica com [123I] MIBG na Denervação Renal
Source: Arq Bras Cardiol. 2022 Feb 14;118(2):519–24. [Article in Portuguese] doi: 10.36660/abc.20201253 (PMC8856689; doi:10.36660/abc.20201253)
Supplement: Translation

# Translation: Risk Stratification and Cardiac Sympathetic Activity Assessment Using Myocardial [^123^I] MIBG Imaging in Renal Denervation

**Authors:** Joana Delgado-Silva, Ana Paula Moreira, Gracinda Costa, Lino Gonçalves

**Affiliations:** 1 Portugal Faculdade de Medicina (FMUC), Universidade de Coimbra, Coimbra – Portugal; 2 Portugal Departamento de Cardiologia, Centro Hospitalar e Universitário de Coimbra (CHUC), Coimbra – Portugal; 3 Portugal Departamento de Medicina Nuclear, Centro Hospitalar e Universitário de Coimbra (CHUC), Coimbra – Portugal; 4 Portugal Instituto de Ciências Nucleares Aplicadas à Saúde (ICNAS), Universidade de Coimbra, Coimbra – Portugal; 5 Portugal Instituto de Investigação Clínica e Biomédica de Coimbra (iCBR), Faculdade de Medicina, Universidade de Coimbra, Coimbra – Portugal

**Keywords:** Resistant Hypertension, Kidney/denervation, Sympathetic Nervous System, Myocardial/diagnostic imaging, Radionuclide Imaging, 3-Iodobenzylguanidine

## Abstract

Hyperactivation of the sympathetic nervous system plays a central role in the pathophysiology of hypertension. The aim of this study was to assess cardiac sympathetic activity and investigate the role of myocardial^123^I-labelled meta-iodo benzyl guanidine ([^123^I] MIBG) scintigraphy in cardiovascular risk stratification of patients with resistant hypertension treated with renal denervation (RDN). Eighteen patients were included in this prospective study (mean age 56 ± 10 years old, 27.8% females). Transthoracic echocardiogram, general blood analysis and myocardial ([123I] MIBG scintigraphy were performed before and six-months after RDN. A patient was considered a responder (R) if a drop ≥ 5mmHg on mean systolic ambulatory blood pressure (BP) monitoring was observed at the six-month follow-up. 66.7% of patients were R (drop in systolic BP of 20.6 ± 14.5mmHg, vs minus 8 ± 11.6mmHg in non-responders (NR), p=0.001). Early heart-mediastinum ratio (HMR) was significantly lower at baseline in the R group (1.6 ± 0.1 vs 1.72 ± 0.1, p<0.02) but similar at six months. Considering both instants in time, the R group had lower early HMR values than the NR group (p<0.05). Both the late HMR and the washout rate were identical and no significant correlation between response to RDN or any MIBG imaging index was found. Renal denervation effectively lowered blood pressure in the majority of patients but [^123^I] MIBG was not useful in predicting the response. However, there was evidence of sympathetic overdrive and, both early and late HMR were overall reduced, probably putting this population at a higher risk of adverse events.

## Introduction

Hypertension (HT) has long been recognized as one of the leading causes of cardiovascular death and hospitalizations.^1^ According to the current guidelines, HT is defined as resistant when optimized pharmacological therapy with three anti-hypertensive drugs, including a diuretic, is unable to effectively lower systolic and diastolic blood pressure (BP) to < 140mmHg and <90mmHg, respectively. Its prevalence is thought to be around 5-15%.^2^ The sympathetic nervous system (SNS) and its involvement in circulatory regulation was first demonstrated in the 19th century by showing that stimulation of renal nerves elevated BP.^3^ According to this knowledge, invasive procedures targeting the SNS were developed in early/mid-20th century but were discontinued, due to increased side effects and mortality.^4^ Since then, clarification of the mechanisms by which the SNS leads to uncontrolled BP conducted to the development of a minimally-invasive percutaneous procedure that has been shown to reduce renal and central sympathetic activity.^5^ Renal denervation (RDN) has been the subject of extensive investigation in the past few years and, the latest second-generation randomized trials have demonstrated both the efficacy in lowering BP, but also safety, in cohorts of patients at different levels of cardiovascular risk.^6123^I-labelled meta-iodo benzyl guanidine ([^123^I] MIBG) is an analogue of norepinephrine (NE), labeled with iodine-123, which shares the same uptake mechanism in pre-synaptic nerves. After the uptake, it is transported to catecholamine storage vesicles and, as it is not metabolized, it allows the characterization of cardiac sympathetic activity and neuronal integrity, through planar imaging acquisition using gamma cameras. By analyzing the images, two semi-quantitative parameters are calculated, early and late heart-mediastinum ratio (HMR) and washout rate (WR). Increases in [^123^I] MIBG concentration in the synaptic cleft translated into augmented WR and diminished HMR.

In the long term, a chronic hyperactivated SNS lead to a significant lack of function/reduction of NE transporters (increasing NE synaptic concentration) and to the exhaustion of NE storage vesicles. The excessive amount of cardiac catecholamines promote fibrosis, cardiomyocyte necrosis and predispose to severe arrhythmic events. The early images obtained with myocardial [^123^I] MIBG scintigraphy (MIBG-S) characterize interstitial uptake, reflecting the integrity of pre-synaptic neurons, while late images represent the distribution of sympathetic nerve terminals, reflecting neuronal function. The WR represents the ability of the myocardium to retain MIBG and it depends on neuronal integrity and degree of sympathetic activity.^7^

The objective of this study was to assess cardiac sympathetic activity and investigate the role of myocardial [^123^I] MIBG-S in cardiovascular risk stratification of patients with resistant HT treated with RDN.

## Methods

This single-center, prospective study included 18 consecutive patients with resistant HT treated with RDN, from May 2014 to October 2017. A comprehensive medical history was recorded for all patients and untreated secondary HT was excluded. Adherence to drug therapy was confirmed by witnessed intake (patients were admitted to the cardiology ward for a 24-hour period). Exclusion criteria included recent major adverse cardiovascular events, fibromuscular dysplasia, previous renal angioplasty, glomerular filtration rate < 45mL/min/1.73m^2^, untreated secondary HT and pseudo-HT (90 patients excluded). Patients with a mean systolic BP >135mmHg (ambulatory blood pressure monitoring – ABPM) were included. A total of 108 patients with suspected true resistant hypertension were evaluated in the outpatient clinic and 90 patients were excluded, according to the described criteria. All patients underwent a thorough clinical evaluation, electrocardiogram, transthoracic echocardiogram, standard hematologic and biochemistry profile, and MIBG-S, both at baseline and at six months follow-up. For the RDN procedure, the EnligHTN multielectrode system (St. Jude Medical, MN, USA) was employed in 33.3% of the cases and the Symplicity Spyral multielectrode catheter (Medtronic Inc., Santa Rosa, CA, USA) in 66.7%. All patients received conscious sedation and analgesia, and femoral artery hemostasis was achieved using a vascular closing device. Before MIBG-S, the patients were pre-treated with Lugol’s solution for thyroid blockade (equivalent to 130 mg of iodine for adults) or 500mg of potassium perchlorate if the patient was allergic to iodine. Afterwards, an intravenous injection of 185 MBq of [^123^I] MIBG was administered, and planar images of the thorax were acquired with a dual-headed gamma camera, fifteen minutes (early imaging) and four hours (late imaging) after the radiopharmaceutical administration. MIBG uptake was semiquantified by calculating the HMR, after drawing ROIs over the heart (including the cavity) and the upper mediastinum (avoiding the thyroid gland) in the planar anterior view. Average counts per pixel in the myocardium were divided by average counts per pixel in the mediastinum. The myocardial WR from the initial to the late images was also calculated, and expressed as a percentage, being the rate of reduction in myocardial counts over time, between early and late imaging (normalized to mediastinal activity). None of the prescribed medications were stopped for the performance of MIBG-S, due to high probability of adverse events and hence ethical issues. Response to RDN was defined if a drop in mean ABPM systolic BP ≥ 5mmHg was observed at six months and patients were divided into two groups accordingly.

Categorical variables were characterized by determining the absolute and relative frequencies, and numerical variables through the means and standard deviations. Normality of distribution was checked and a p value of <0.05 was considered significant. Comparisons between groups with regard to the categorical variables were conducted using the Chi-Square Test. Regarding the continuous variables, the Mann-Whitney U Test was used to compare two groups. A general linear model for repeated measures was applied to analyze variance of each parameter, measured before and after RDN in each subject from two different groups, i.e., ‘responder’ and ‘non-responder’. Statistical analyses were conducted using SPSS 19.0®, at a 5% significance level for hypothesis-testing. This study was approved by the Coimbra’s Faculty of Medicine Ethics Committee and all patients signed an informed consent form.

## Results

Eighteen patients (mean age 56 ± 10 years old, 27.8% female gender) were included in this study. Twelve patients were ‘responders’ (R, 66.7%) and six ‘non-responders’ (NR, 33.3%). No significant differences were observed between the groups regarding baseline characteristics. RDN was well tolerated by all patients and no peri-procedural complications were detected. Fluoroscopy time was significantly higher in the NR group (16.3 ± 5.5 vs 26.5 ± 18.6 minutes, p<0.04). At the 6-month follow-up, one patient had an acute pulmonary edema, being diagnosed with renal stenosis, successfully treated with angioplasty. This patient was a ‘responder’ as a ≥ 5mmHg drop in mean ABPM systolic BP was observed 15 days after the angioplasty. A drop of 20.6 ± 14.5mmHg in mean ABPM systolic BP was observed in the R group (vs -8 ± 11.6mmHg in NR, p=0.001). Even though office systolic BP was not considered for the response, due to a possible ‘white-coat effect’, a drop was also observed in the R group (29.2 ± 8.4mmHg) vs the NR group (13 ± 13.4mmHg) (p=0.09). No side effects such as orthostatic hypotension, electrolyte disturbances or renal failure were noticed in the medium-term follow-up. The findings in transthoracic echocardiography (regarding diastolic function, wall thickness or biventricular systolic function) did not differ significantly between the two groups, either at baseline or after 6 months assessment. Baseline and procedural-related characteristics of the overall, ‘responders’ and ‘non-responders’ groups, are shown in table 1 .

**Table 1:** Baseline and procedural-related characteristics, ABPM baseline and 6 months evolution and MIBG scintigraphy parameters at baseline and at 6-month follow-up, in the overall, ‘responder’ and ‘non-responder’ groups

	Overall	R (n=12)	NR (n=6)	p value
**General baseline features**				
Mean age (Y)	56 ± 10	58.4 ± 9.8	51.3 ± 10.3	0.17 (ns)
Female gender (%)	27.8	16.7	50	0.14 (ns)
Diagnosis of HT (Y)	19 ± 7.9	19.8 ± 8.7	17.5 ± 6.2	0.57 (ns)
Dyslipidemia (%)	88.9	83.3	100	0.29 (ns)
Diabetes (%)	44.4	41.7	50	0.73 (ns)
Active smoking (%)	27.8	16.7	50	0.14 (ns)
BMI (Kg/m2)	29.7 ± 4.1	29.5 ± 4	30 ± 4.7	0.84 (ns)
Sleep apnea (%)	66.7	66.7	66.7	1 (ns)
Number of HT drugs (n±SD)	5.2 ± 1.2	5.2 ± 1.5	5.3 ± 0.5	0.79 (ns)
Spironolactone (%)	61.1	66.7	50	051 (ns)
Calcium channel blockers (%)	100	100	100	----
Beta-blockers (%)	77.8	75	83.3	0.7 (ns)
ACE inhibitors/ARBs (%)	94.4	91.7	100	0.48 (ns)
Diuretics (%)	94.4	91.7	100	0.48 (ns)
Alpha2-blockers (%)	61.1	58.3	66.7	0.74 (ns)
Baseline Creatinine (mg/dL)	0.88 ± 0.2	0.9 ± 0.2	0.7 ± 0.2	0.56 (ns)
**Baseline and 6M Echo**				
Baseline ejection fraction (%)	59 ± 9	59 ± 9	59 ± 8	0.94 (ns)
6M ejection fraction (%)	58 ± 9	56 ± 9	62 ± 9	0.21 (ns)
Baseline IVS thickness (mm)	12.4 ± 3.7	13.4 ± 4.2	10.5 ± 1.4	0.12 (ns)
6M IVS thickness (mm)	12.9 ± 2.6	13.4 ± 3	12 ± 1.3	0.29 (ns)
Baseline PW thickness (mm)	10.9 ± 1.9	11.5 ± 2	9.7 ± 1.2	0.06 (ns)
6M PW thickness (mm)	10.2 ± 2.3	10.5 ± 2.5	9.7 ± 1.6	0.48 (ns)
Baseline LA volume (ml/m^2^)	56.4 ± 19.5	53.8 ± 14.8	61.8 ± 11.3	0.43 (ns)
6M LA volume (ml/m^2^)	56 ± 22.3	59 ± 23	51 ± 22	0.51 (ns)
Baseline E/E’	11.1 ± 3.5	9.9 ± 3	13.6 ± 3.2	0.02
6M E/E’	11 ± 3.5	10.9 ± 4	11 ± 2.4	0.95 (ns)
**Baseline and 6M ABPM**				
Baseline mean SBP (mmHg)	154.6 ± 11.7	154.5 ± 11.4	154.8 ± 13.5	0.96 (ns)
Drop in SBP 6M (mmHg)	11 ± 19.2	20.6 ± 14.5	-8 ± 11.6	0.001
Baseline mean DBP (mmHg)	90.7 ± 14	88.2 ± 13.5	95.7 ± 14.8	0.29 (ns)
Drop in DBP 6M (mmHg)	6.3 ± 9	10.4 ± 7.1	-1.8 ± 6.5	0.004
Baseline heart rate (bpm)	71 ± 10	70 ± 9	73 ± 14	0.67 (ns)
6M heart rate	70 ± 10	68 ± 9	76 ± 11	0.44 (ns)
**Renal denervation**				
Number of ablations (n ± SD)	27.2 ± 7.7	28.7 ± 8.1	24.2 ± 6.3	0.25 (ns)
Fluoroscopy time (min)	19.3 ± 11.4	16.3 ± 5.5	26.5 ± 18.6	<0.04
**Cardiac MIBG scintigraphy**				
Baseline HMR 15 min	1.63 ± 0.11	1.59 ± 0.10	1.72 ± 0.08	<0.02
6M HMR 15 min	1.64 ± 0.12	1.61 ± 0.10	1.70 ± 0.14	0.14 (ns)
Baseline HMR 4 hours	1.60 ± 0.11	1.59 ± 0.10	1.64 ± 0.14	0.22 (ns)
**Continuation**				
6M HMR 4 hours	1.60 ± 0.16	1.59 ± 0.12	1.63 ± 0.24	0.64 (ns)
Baseline WR	22.7 ± 18.6	17.9 ± 10	32.2 ± 28.2	0.13 (ns)
6M WR	25.9 ± 16.4	25.4 ± 17.9	27 ± 14.2	0.86 (ns)

*Y: years; HT: hypertension; BMI: body mass index; ACE: angiotensin-converting enzyme; ARBs: angiotensin receptor blockers; M: months; IVS: intraventricular septum; PW: posterior wall; LA: left atrium; ABPM: ambulatory blood pressure monitoring; SBP: systolic blood pressure; DBP: diastolic blood pressure; bpm: beats per minute; MIBG:^123^I-labelled metaiodobenzylguanidine; HMR: heart-mediastinum ratio; WR: washout rate. Results are displayed in mean ± standard deviation (SD).*

Early HMR was significantly lower at baseline in the R group (1.6 ± 0.1 vs 1.72 ± 0.1, p<0.02, 95% CI 1.6-1.71) but was not statistically different from the NR group at six months. Putting together both times periods, the R group had lower early HMR values than the NR group (p<0.05, 95% CI 1.58-1.69). Regarding late HMR and WR, differences before and after RDN were not significant between groups. No significant correlation between response to RDN or any [^123^I] MIBG imaging index was found, either at baseline or at the follow-up ( table 1 , figure 1 and supplementary table 1 ).

**Figure 1:** – Myocardial MIBG scintigraphy in patients submitted to renal denervation (RDN). (A) Early (15 minutes) and late (4 hours) heart-mediastinum ratio (HMR) at baseline and six-months (6M) after RDN, in ‘responders’ (R) vs ‘non-responders’ (NR) – early HMR was significantly lower in R, at baseline; (B) Washout rate at baseline and six-months after RDN in R vs NR; (C) and (D) MIBG scintigraphy, thorax anterior projection, at baseline, in a responder, at 15 minutes (C) and at four hours (D).

## Discussion

The aim of our study was to determine whether RDN had any impact on myocardial sympathetic activity, and also to assess procedure safety, as a significant subsequent decrease in HMR could signify disruption of the sympathetic pathway. We verified a significant reduction in BP six months after RDN, in 66.7% of the patients, which is aligned with the reported efficacy of the technique. No safety issues were reported, except for one patient who was diagnosed with renal artery stenosis six months after the RDN, probably due to radiofrequency delivery next to a non-significant atherosclerotic plaque. We determined that responders had a significantly lower baseline early HMR, which could be due to decreased neuronal integrity, but no significant changes were observed after six months.

Late HMR was similar in both groups but reduced in comparison to values reported in normal subjects (normal reported values 2.2 ± 0.3,^5^ local reference values 1.9-2.8), both at baseline and at the six-month follow-up, translating into a maintained sympathetic hyperactivity even after RDN, and probably being associated with a higher risk of events. WR was also statistically similar in both groups. However, WR was significantly increased overall, in comparison to normal individuals (normal reported mean values 10 ± 9%,^5^ local reference values 8.5-9.6%), with this discrepancy being more evident in non-responders at baseline, due to a possible sympathetic overdrive.

The SNS is an extremely complex system, with clinical implications in both physiological and pathological states. It is characterized by multiple levels of action that involve central regulation, ganglionic transmission, release and reuptake of norepinephrine and the response of adrenergic receptors.^8^ As such, a precise method to evaluate global and regional sympathetic activity does not exist, with each technique having its strengths and limitations.

The effect of RND on sympathetic activity has been previously described. Krum et al.^9^ reported a 47% decrease in the release of noradrenaline from the renal sympathetic nerves bilaterally after RDN, using the isotope dilution renal noradrenaline spillover method. MIBG-S has been performed in small cohorts of RDN patients in order to assess sympathetic activity, but results have been rather divergent, reporting decreases in WR,^5^ increases in late HMR^10^ or no change at al.^11^ This imaging method has also been considered useful to evaluate cardiac sympathetic activity in the context of heart failure, being able to estimate both prognosis and response to treatment. Indeed, in the ADMIRE-HF trial, a significant lower event and cardiac death rate was observed in patients with a late HMR ≥ 1.6.^12^

What is not clear in our study is, given that non-responders had evidence of increased SNS activity, why didn’t they clinically respond to RDN? Were there other factors/systems superseding the contribution of the SNS in the pathophysiology of HT? Furthermore, none of the evaluated rates altered significantly at follow-up, translating into an absence of deleterious sympathetic nerve disruption and, none of the evaluated MIBG parameters were useful to predict response to RDN.

### Limitations

Our study has some limitations. First, this a single-center study and the number of patients enrolled is small. Second, there was no control group. Third, two different denervation systems were used, even though they were both multielectrode. Finally, the study was not randomized and, even though the nuclear medicine specialist was highly experienced, there was no internal or external validation of the results.

## Conclusions

In this study we demonstrate that renal denervation significantly reduced blood pressure in a significant percentage of patients, but there was no evidence of reduced cardiac sympathetic activity visible by myocardial [^123^I] meta-iodine benzyl guanidine scintigraphy. None of the imaging parameters were useful to predict response to renal denervation. However, both early and late heart-mediastinum ratio were found to be reduced/lower, compared to the general population, probably putting this population at a higher risk of events. Large-scale studies are needed to determine the validity of this method in the evaluation of cardiac renal denervation effects.

## * Supplemental Materials

For additional information, please click here .
